# Zebrafish Models for Dyslipidemia and Atherosclerosis Research

**DOI:** 10.3389/fendo.2016.00159

**Published:** 2016-12-16

**Authors:** Amnon Schlegel

**Affiliations:** ^1^University of Utah Molecular Medicine Program, School of Medicine, University of Utah, Salt Lake City, UT, USA; ^2^Department of Internal Medicine, Division of Endocrinology, Metabolism and Diabetes, University of Utah, Salt Lake City, UT, USA; ^3^Department of Biochemistry, School of Medicine, University of Utah, Salt Lake City, UT, USA; ^4^Department of Nutrition and Integrative Physiology, College of Health, University of Utah, Salt Lake City, UT, USA

**Keywords:** atherosclerosis, dyslipidemia, zebrafish, genetics, physiology and metabolism

## Abstract

Atherosclerotic cardiovascular disease is the leading cause of death. Elevated circulating concentrations of lipids are a central pathogenetic driver of atherosclerosis. While numerous effective therapies for this condition have been developed, there is substantial unmet need for this pandemic illness. Here, I will review nutritional, physiological, genetic, and pathological discoveries in the emerging zebrafish model for studying dyslipidemia and atherosclerosis. The technical and physiological advantages and the pharmacological potential of this organism for discovery and validation of dyslipidemia and atherosclerosis targets are stressed through summary of recent findings. An emerging literature shows that zebrafish, through retention of a *cetp* ortholog gene and high sensitivity to ingestion of excess cholesterol, rapidly develops hypercholesterolemia, with a pattern of distribution of lipid species in lipoprotein particles similar to humans. Furthermore, recent studies leveraging the optical transparency of zebrafish larvae to monitor the fate of these ingested lipids have provided exciting insights to the development of dyslipidemia and atherosclerosis. Future directions for investigation are considered, with particular attention to the potential for *in vivo* cell biological study of atherosclerotic plaques.

## Introduction

Atherosclerosis is the leading cause of death (1). This chronic, progressive build-up of cholesterol, cellular debris, and calcium can narrow the lumens of critical arteries supplying the heart, brain, limbs, and organs. Plaques are mechanically weak structures and are prone to rupture. Once ruptured, a rapid thrombosis cascade is activated at the site of the plaque, occluding the artery and causing ischemic death to the supplied organ. Persons who have sustained an ischemic event in any artery are at substantially increased risk of repeated plaque rupture and thrombosis. While the last several decades have witnessed a decrease in the incidence of myocardial infarction and ischemic cerebrovascular accident, cardiovascular death is predicted to remain the leading killer for decades to come. A confluence of cardiovascular risk factors including tobacco exposure, hypertension, obesity in children and adults, type 2 diabetes mellitus, and non-alcoholic fatty liver disease is to blame for this trajectory (2–6). Moreover, there is widespread underutilization of effective antiplatelet, antihypertensive, and lipid lowering therapies (3, 7).

In the face of this clinical reality, I will argue in this minireview that zebrafish is an excellent system to discover and characterize new diagnostic and therapeutic targets for atherosclerosis. Those properties that make the study of lipid physiology and atherosclerosis in zebrafish potentially transformative will be reviewed, with an emphasis on original work as throughout the remainder of this article, stressing studies published since others and I last reviewed this topic (8, 9).

## Zebrafish Model Overview

The general strengths of zebrafish for biomedical research are well known, owing to its facile husbandry and low cost of housing and maintenance. This organism can be used to generate large numbers of externally fertilized embryos. These animals develop rapidly and are a mainstay of embryological, forward genetic, and pharmacological research (10–12). In the last decade, a full array of modern genome editing tools has been deployed in zebrafish, including very promising knock-in technologies (13–15). These advances have been married to progress in working in zebrafish late larvae, juveniles, and adults, where numerous aspects of physiology pertinent to atherosclerosis emerge.

## General Features of Lipoprotein Metabolism in Zebrafish

### Lipoprotein Biology in Zebrafish

**Figure 1:**
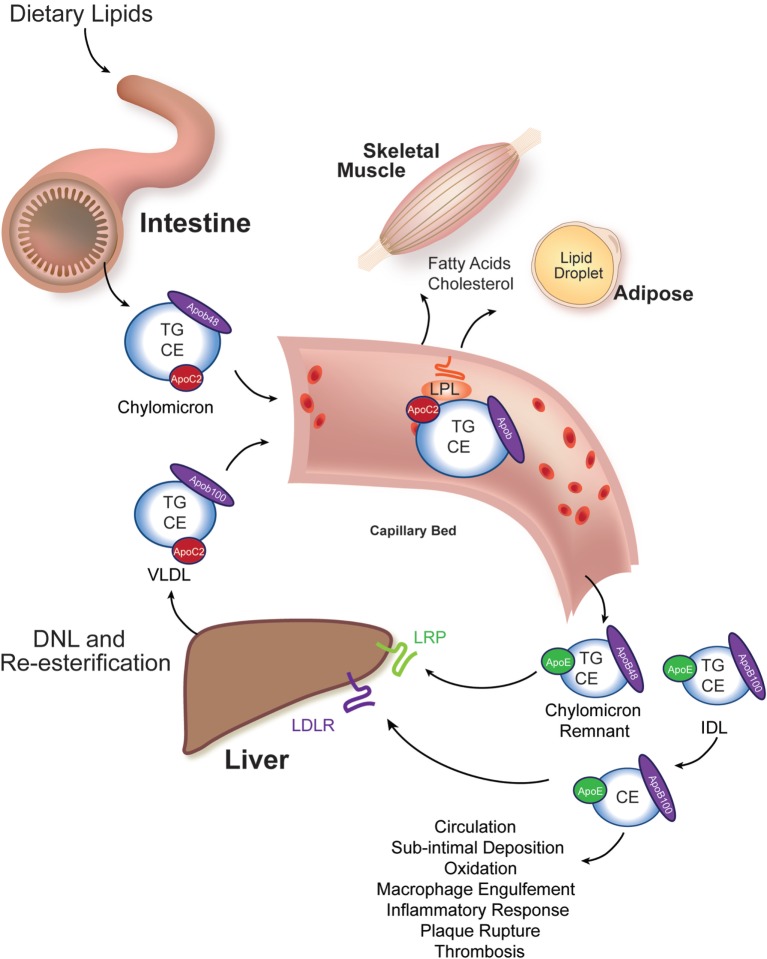
**Intestinal and liver β-lipoprotein synthesis and vascular modification**. Ingested lipids are hydrolyzed in the lumen of the intestine to absorbable species, such as free cholesterol, free fatty acids, and monoacylglycerol. These molecules are re-esterified in the enterocyte of the small intestine to triacylglycerol (TG), cholesteryl esters (CE), and phospholipids (not shown) and packaged into chylomicrons, whose signature coat protein in humans is Apob48 (one molecule per particle). This particle enters the vasculature and acquires an Apoc2 molecule from an HDL particle (not shown). Apoc2 is a required binding partner for lipoprotein lipase (LPL), an enzyme tethered to the apical surface of capillary bed endothelial cells in muscle and adipose tissues (20). LPL liberates free fatty acids for use by these tissues. The partially lipid-depleted chylomicron remnant is rapidly cleared by the liver through the action of Apoe-binding LRP receptors and Apob-binding low-density lipoprotein receptors (LDLR). The liver synthesizes very low-density lipoprotein (VLDL) particles from *de novo* lipogenesis-derived fatty acids and re-esterified fatty acids that reach the liver after adipocyte hydrolysis (and has relatively less CE in it). Human VLDL’s signature coat protein is Apob100. Following LPL-catalyzed lipid hydrolysis, VLDL remnants, intermediate density lipoprotein (IDL) particles, are either rapidly cleared by the liver or mature into LDL. LDL particles have a long circulating half-life, and they can deposit under vascular endothelial cells, undergo oxidation, and trigger an inflammatory atherosclerotic reaction with subsequent plaque rupture and thrombosis causing ischemia to the supplied tissue.

Elevated serum cholesterol and non-fasting triacylglycerol (TG) are central drivers of atherosclerosis (16–18). Understanding how lipids are absorbed from the diet, metabolized in tissues, and modified in atherosclerosis are central areas of investigation in developing newer and more effective therapies to treat atherosclerosis. A highly conserved system for transporting water-insoluble lipids is present in all animals (19). In particular, the apolipoprotein B (APOB)-coated particles produced by the intestine (chylomicrons) and liver [very low-density lipoprotein (VLDL) particles] are the carriers of the bulk of absorbed and resynthesized neutral lipids, cholesteryl esters (CE), and TG. These “β-lipoprotein” particles also carry fat-soluble vitamins A, D, and E from their sites of absorption or synthesis to their sites of use or storage (Figure 1).

In amniotes, two different protein products are encoded by a single *APOB* locus. In enterocytes of reptiles, birds, and mammals, the *APOB* pre-mRNA undergoes cytosine deamination (catalyzed by APOBEC) to generate a transcript encoding a truncated protein (APOB48) that is found exclusively on chylomicrons (21). The full-length *APOB* transcript can be translated in both the liver and intestine (encoding APOB100). APOB48-coated chylomicron remnants are susceptible to rapid postprandial clearance by the liver, whereas APOB100-coated chylomicron remnants and VLDL remnants [intermediate density lipoprotein (IDL) particles] can mature into the long-lived and atherogenic low-density lipoprotein (LDL) particles (Figure 1). Thus, it is important to appreciate that Apob (operationally equivalent to “Apob100”)-coated zebrafish chylomicrons are, most likely, not cleared rapidly. Furthermore, zebrafish chylomicrons carry the potential to mature into LDL stoichiometrically (22). This lack of Apob48 might contribute to the rapid dyslipidemia and atherogenesis seen in dietary and genetic studies of zebrafish that will be discussed in subsequent sections. Finally, there are two zebrafish *apob* paralogs (two *apob* genes on different chromosomes). The contribution (expression and incorporation into chylomicrons and VLDL) of these Apob paralogs to circulating β-lipoproteins and atherogenesis is not known; however, their larval expression patterns are different, and their encoded proteins are structurally dissimilar, raising the possibility that they have unique functions (23).

Immediately beyond these critical issues of Apob biology, zebrafish utilizes highly conserved β-lipoprotein assembly proteins and transport mechanisms. Gene expression survey and knockdown approaches confirmed that the central Apob-coated lipoprotein particle-producing enzyme microsomal triglyceride transfer protein (encoded by *mtp* and having orthologs in all species ranging from insects to mammals) is present and functional in zebrafish yolk cell layer, liver, and intestine (24–26). More recently, studies on the intracellular trafficking of nascent chylomicrons have confirmed that the zebrafish model is well suited to investigating the molecular and cellular machinery of dietary energy harvest: the enterocyte undergoes stereotypical changes in ultrastructure when absorbing fats, and its secretory apparatus uses proteins conserved in evolution to pack and traffic nascent chylomicrons (27, 28). Finally, the major determinant of clearance of LDL particles from the circulation, the LDL receptor (Ldlr), has conserved function in zebrafish (29). In short, zebrafish has a complement of conserved lipid trafficking genes that renders study of lipid transport in this model organism relevant to human physiology. The next section will consider one additional, critical circulating protein that makes zebrafish lipoprotein biology particularly useful for modeling human lipoprotein biology.

### Cholesteryl Ester Transfer Protein (CETP)

**Figure 2:**
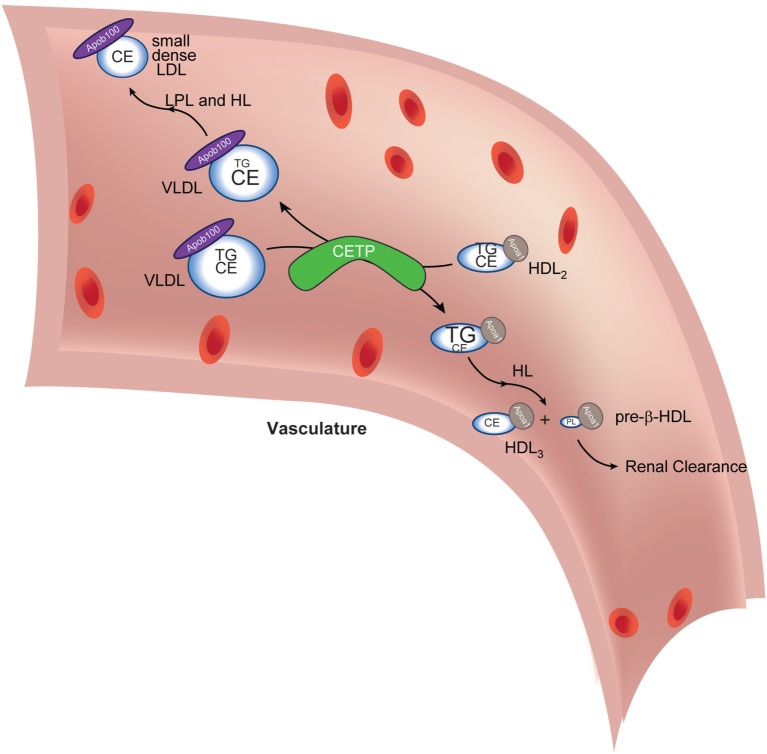
**Cholesteryl ester transfer protein (CETP) in lipoprotein lipid exchange**. Very low-density lipoprotein (VLDL) particles and HDL_2_ exchange triacylglycerol (TG) and cholesteryl esters (CE) in a reaction catalyzed by CETP. The depletion of TG and increase in VLDL CE (reflected in altered font sizes) coupled with lipoprotein lipase (LPL)- and hepatic lipase (HL)-mediated (further) depletion of TG (not shown) lead to the formation of small dense low-density lipoprotein (LDL), which is amenable to oxidative modification, a conversion central to driving subsequent atheromatous plaque formation. The transient increase in TG in HDL_2_ (reflected in increased font size) delivers a substrate for HL-mediated hydrolysis (as it passes through the liver capillaries). This reaction generates small HDL_3_ and pre-β-HDL, which contain scant amounts of phospholipids only. Pre-β-HDL is removed from the circulation *via* renal filtration. The net effect of CETP action, thus, is to cause maturation of VLDL into atherogenic, small, dense LDL and to decrease atheroprotective HDL concentration. Apoa1 is the signature coat protein of HDL.

Following release into the circulation, lipoproteins are modified in zebrafish blood by enzymatic machinery that is also highly conserved with humans. Specifically, zebrafish carries an ortholog of the human *CETP* gene (30). CETP encodes a circulating protein that transfers CE from HDL particles to LDL particles in exchange for TG (Figure 2). Once loaded with TG and subject to additional modification, HDL is rendered more prone to rapid clearance, decreasing its “ability” to engage in atheroprotective processes such as reverse cholesterol transport (i.e., retrieving cholesterol from tissue macrophages to delivery to the liver and intestine for elimination). Likewise, increased CE loading of and depletion of TG from LDL contribute to atherogenesis by producing readily modifiable (oxidizable) small dense particles that can enter the subintimal space and drive atherogenesis (31–33). The net effect of Cetp function is to leave the organism with a higher concentration of atherogenic LDL particles and a lower concentration of atheroprotective HDL particles in circulation (the so-called “β-dominant” lipoprotein profile). In zebrafish, the fasting lipoprotein profile is β-dominant (34). This similarity to human lipoprotein composition reflects retention of a *cetp* ortholog in the zebrafish genome. As discussed below, this conservation of a critical human lipoprotein-modifying enzyme renders zebrafish susceptible to a dyslipidemia with short dietary interventions. This conservation of a critical aspect of lipoprotein biology also opens the door to pharmacological intervention studies in zebrafish: many other, non-rodent laboratory models also fall short of recapitulating human lipoprotein composition and atherogenesis (35).

A Cetp ortholog is absent in commonly used rodent models of dyslipidemia, rendering the study of atherosclerosis inherently difficult in these species. Specifically, rodents are resistant to atherosclerosis because, among other things, they lack this enzyme’s action. Rodents have high circulating HDL concentrations and low LDL concentrations (the so-called “α-dominant” lipoprotein pattern) as a consequence of losing the *Cetp* gene (35). The most commonly used genetic strategy to trigger dyslipidemia in mice is to study dietary and genetic interventions in the context of deleting the *Apoe* or *Ldlr* genes (36–40). Although they are widely used, these models do not capture the full biology of the corresponding human Mendelian diseases, familial dysbetalipoproteinemia (in the case of *Apoe*), and familial hypercholesterolemia (in the case of *Ldlr*): the HDL-cholesterol in both *Apoe*^−/−^ or *Ldlr*^−/−^ mice is still higher than in humans with *APOE^2^*^/^*^2^* or *LDLR*^−/−^ genotypes, and in both models, it is mainly the VLDL (and not the IDL and LDL, respectively) that increases. Furthermore, studies with only *Apoe*^−/−^ or *Ldlr*^−/−^ mouse models are often limited in generalizability: there is incomplete agreement in the findings with these two models (41). Beyond the limitations of standard mouse genetic models in driving atherogenic dyslipidemia, the atheromatous plaques that do form in mice lack features of “complex” human lesions. Indeed, to generate atheromatous plaques that appear more like human plaques, extreme physical stress is required in *Apoe*^−/−^ animals deliberately maintained on a mixed genetic background (42). Even with this severe stress, plaque rupture (as in myocardial infarction) does not occur in rodents. Another supraphysiological approach to studying plaque rupture in mice that has met with some criticism because of its artificial nature involves causing a prolonged pharmacological hypertensive crisis in *Apoe*^−/−^ animals; this paradigm causes plaque rupture in the brachiocephalic artery, an uncommon site of rupture in humans (43).

## Zebrafish Dyslipidemia Models

### High-Cholesterol Diet (HCD) Paradigm

The nutritional requirements of zebrafish are now known (44, 45). This knowledge has allowed several groups to establish conditions to induce metabolic stress by altering standard diets. For instance, zebrafish is susceptible to high-fat diet (HFD)-induced obesity, hyperglycemia, and dyslipidemia (46). The major breakthrough in applying zebrafish to the study of dyslipidemia was the development of HCD (34, 47, 48). Not only do larvae and adults readily ingest such diets but also these animals demonstrate a series of responses that firmly established the utility of this organism in studying dyslipidemia and atherogenesis. First, HCD-challenge caused β-dominant hypercholesterolemia in adults; second, vascular intimal lipid accumulation can be seen in larvae after short exposure to HCD, and these accumulated lipids attracted circulating monocytes; third, the extravasated LDL undergoes oxidation (to generate high-affinity ligands for innate immune receptors that are central for driving the inflammation of plaques); and fourth, the oxidized LDL particles can be tracked with live imaging (34, 47, 48). This last observation was made *via* transgenic overexpression of the human monoclonal antibody IK17, which binds to malondialdehyde-modified LDL. The sustained overexpression of IK17 prevented HCD-induced sub-intimal lipid accumulation (47). This is the first proof-of-principle demonstration that atherogenesis can be prevented in zebrafish through, presumably, accelerating immune complex-mediated clearance of modified LDL particles from the circulation (before they deposit in the walls of arteries). This constellation of findings sets the stage for future live imaging of atheromas *in vivo*, as discussed below.

### CETP Pharmacology

Natural compound extracts of cinnamon, clove, grape skin, laurel, loquat, and turmeric contain inhibitors of zebrafish Cetp (30, 49, 50). These extracts protect zebrafish from high-cholesterol diet-induced dyslipidemia. Conversely, artificial sweeteners increase HDL particle-carried Cetp activity and drive hyperlipidemia (51, 52). Whether such zebrafish studies will translate into better inhibitors of human, CETP is difficult to predict; moreover, artificial sweeteners appear to exert multiple pathological effects, including triggering glucose intolerance by altering the gut microbiome (53). This pharmaceutical research space has been marked by several abandoned small molecules; one ongoing cardiovascular outcomes trial of a CETP inhibitor (http://ClinicalTrials.gov identifier NCT01252953) and a possible study of another inhibitor might provide opportunities for not only using this approach in humans but also to identify additional questions that might be answered with a zebrafish model (54, 55).

### Zebrafish APOC2 Deficiency

A further advance in developing zebrafish dyslipidemia models comes from the targeted deletion of the *apoc2* gene. Humans lacking APOC2 have familial chylomicronemia, a condition marked by high serum TG concentrations and propensity to recurrent bouts of pancreatitis (56). Zebrafish *apoc2^−^*^/^*^−^* mutants were generated with genome editing tools (22). These mutants demonstrated the hallmark finding of human APOC2 deficiency: decreased plasma lipase activity and severe hypertriglyceridemia (Figure 1). Their lipoprotein pattern is predominantly large, β-lipoproteins, as quantified with size-exclusion chromatography and scanning electron microscopy techniques. Imaging of the vasculature in *apoc2^−^*^/^*^−^* mutants reveals accumulation of lipids and lipid-laden macrophages, both hallmarks of atherosclerotic plaques. This powerful dyslipidemia model might prove particularly useful in studying the steps of LDL extravasation, oxidation, and engulfment by vascular wall macrophages.

### Zebrafish Liver X Receptor (LXR) Deletion

Lxrs are central inducers of cholesterol catabolism (57). These nuclear receptor transcription factors regulate metabolism through engaging oxysterol ligands and altering expression of functionally integrated genes involved in reverse cholesterol transport, lipoprotein modification, intestinal cholesterol absorption and excretion, liver fatty acid and TG regulation, bile transport, and immune and inflammatory signaling (57). There are two Lxr paralogs in mammals. Lxrα, which arose in fish, is mainly expressed in tissues involved in tissue macrophages, liver, and intestine, whereas Lxrβ, which arose in amphibians, is more widely expressed (58). Lxrα upregulates hepatic lipogenic enzymes and increases blood TG levels (59, 60). This seemingly self-defeating function—driving elimination of cholesterol while triggering fatty acid synthesis—has been a major impediment to developing Lxr-based therapeutics. Indeed, the LXRβ-selective agonist BMS-852927 not only promotes reverse cholesterol transport in humans but also induces hepatic *de novo* lipogenesis and attendant hypertriglyceridemia; BMS-852927 also causes a rapid decrease in circulating neutrophil counts in humans, but not in cynomolgus monkeys, underscoring the challenge of drug development (61).

Zebrafish bearing a targeted deletion mutation of the Lxrα gene *nr1h3* develop severe hypercholesterolemia and hepatic steatosis when fed HCD and HFD (62). Conversely, overexpression of *nr1h3* in enterocytes confers protection from dyslipidemia and hepatic steatosis when animals are fed a HFD; this metabolically beneficial effect of *nr1h3* overexpression is due to the induction of a transcriptional program resulting in temporary enterocyte storage of lipids, delaying an *en masse* delivery of atherogenic lipoprotein particles in the circulation. As noted above, zebrafish chylomicrons likely mature into LDL particles because of their full-length Apob-coat protein. As such, the *nr1h3* gene deletion and intestine-limited overexpression models might be useful for studying atherogenesis in that the increase in LDL-cholesterol seen in *nr1h3^−^*^/^*^−^* mutants is substantial. Furthermore, these studies may lead to the rational development of intestine-limited LXR agonists to blunt the development of dyslipidemia and atherosclerosis.

## The Future: Atheroma Cell Biology, Genetic and Pharmacological Screens, and Candidate Gene Analyses

### Cell Biology of Atherosclerosis *In Vivo*

There is no *a priori* guarantee that zebrafish atheromas will model the full natural history of the human disease more closely in terms of mechanisms of development, inflammatory response, and final architecture than available models. However, if the advances in studying lipoprotein biology are any guides, the natural history of atheroma progression—from simple lipid accumulation below the vasculature to organization into “complex,” cell-rich, and debris-rich plaques—should be feasible in zebrafish. In particular, live fluorescent markers of various cell types that accumulate within plaques such as vascular smooth muscles, macrophages, and other immune cells are available. These live imaging reporters could be used to monitor atherogenesis in real time. Notably, such live imaging tools have been deployed with remarkable success in studying architecturally complex mycobacterial infection and host response in zebrafish (63–65). This success in modeling a human host response to mycobacteria in zebrafish—where other laboratory species have fallen short of producing human-like granulomas—is cause for hope that zebrafish atherosclerosis will reveal conserved inflammatory and immune mechanisms. In particular, the observation that zebrafish macrophages form granulomas in response to mycobacterial infection raises the hope that these cells may very well form lipid-laden “foam cells” in atherosclerotic plaques and, thus, drive an evolutionarily conserved inflammatory response that defines “complex” lesions (i.e., recruiting additional cells to the plaque and driving inflammation). Whether zebrafish will be useful in studying all aspects of more advanced atherosclerotic plaques biology is not clear. For instance, zebrafish have much lower blood pressure than terrestrial animals. Whether this organism will be useful for generating plaque rupture models is difficult to predict. Systematic histological examination of zebrafish arteries from dyslipidemia models will be required to determine what aspects of plaque biology can be studied in this model.

### Genetic and Chemical Screens for Dyslipidemia and Atherosclerosis Modifiers

Beyond hemodynamic concerns regarding the natural history of zebrafish atherosclerosis, it remains uncertain whether genetic or pharmacological screens could be designed to look for modulators of dyslipidemia and atherogenesis in zebrafish. These complex phenotypes develop after a period of feeding in late larvae, raising the time and effort required to perform a large-scale screening project. Developing convenient reporters for the development of dyslipidemia and atherogenesis and their validation in already established zebrafish models would help guide screen design. Such an approach has proven feasible and informative in studying fasting glucose regulation in larvae (66, 67).

### Candidate Genes

A large repertoire of genetic loci has been associated on a population genetics level with lipid parameters (68). For most, the molecular and cellular bases of the associations are unknown. While it is now experimentally tractable to rapidly overexpress and delete genes in zebrafish, phenotypic characterization for atherosclerosis is still limited in comparison to examination of alterations in glucose metabolism (for which larvae phenotypes develop more rapidly and do not require feeding). For instance, my group recently explored the mechanistic association of a single nucleotide polymorphism (SNP) in the *FOXN3* locus with fasting blood glucose (69). Through a blend of primary human hepatocyte, immortalized HepG2 hepatoma cell, and transgenic zebrafish approaches, we found that this SNP increases the expression of the FOXN3 protein and that this transcriptional repressor blunts a glucose utilization transcriptional program in the liver. Overexpression of both human *FOXN3* and zebrafish *foxn3* in liver increased fasting blood glucose in adults. Whether this gene acts in other tissues to modulate blood glucose is not known. Neither is the mechanism through which the risk allele increases FOXN3 expression. An approach similar to the one used to study FOXN3 could be used to examine the effect of overexpressing candidate genes (in liver or elsewhere) on zebrafish lipoprotein metabolism, again, with the caveat that the experimental window will need to be larger. Those “hits” showing changes in circulating lipids could be explored further using a large collection of null alleles (70) or through genome editing approaches (including conditional alleles).

## Conclusion

Through a combination of genetic, developmental, and physiological advantages, the zebrafish has emerged as a major mechanistic discovery platform for studying dyslipidemia and atherosclerosis. Here, I highlighted individual areas of success using this organism, from dietary interventions, exploration of various aspects of lipoprotein production and processing, and genetic models for dyslipidemia and early atherosclerosis. Future work in the zebrafish system should include more thorough exploration of the composition and cellular architecture of atheromatous plaques, screenings effort to identify novel genes and small molecules that modulate atherogenesis, and candidate gene approaches to elucidating the functions of loci implicated in dyslipidemia and atherosclerosis through population genetic analyses. Collectively, this work in zebrafish may lead to the development of new and more effective therapies for dyslipidemia and atherosclerosis.

## Author Contributions

The author is the sole contributor to the conceptual development and writing of this minireview.

## Conflict of Interest Statement

The author declares that the research was conducted in the absence of any commercial or financial relationships that could be construed as a potential conflict of interest.

## References

[1] Lozano R Naghavi M Foreman K Lim S Shibuya K Aboyans V Global and regional mortality from 235 causes of death for 20 age groups in 1990 and 2010: a systematic analysis for the Global Burden of Disease Study 2010. Lancet (2012) 380(9859):2095–128. 10.1016/S0140-6736(12)61728-0 23245604 PMC10790329

[2] Mathers CD Loncar D. Projections of global mortality and burden of disease from 2002 to 2030. PLoS Med (2006) 3(11):e442. 10.1371/journal.pmed.0030442 17132052 PMC1664601

[3] Danaei G Finucane MM Lin JK Singh GM Paciorek CJ Cowan MJ National, regional, and global trends in systolic blood pressure since 1980: systematic analysis of health examination surveys and epidemiological studies with 786 country-years and 5.4 million participants. Lancet (2011) 377(9765):568–77. 10.1016/S0140-6736(10)62036-3 21295844

[4] Ng M Fleming T Robinson M Thomson B Graetz N Margono C Global, regional, and national prevalence of overweight and obesity in children and adults during 1980-2013: a systematic analysis for the Global Burden of Disease Study 2013. Lancet (2014) 384(9945):766–81. 10.1016/S0140-6736(14)60460-8 24880830 PMC4624264

[5] Danaei G Finucane MM Lu Y Singh GM Cowan MJ Paciorek CJ National, regional, and global trends in fasting plasma glucose and diabetes prevalence since 1980: systematic analysis of health examination surveys and epidemiological studies with 370 country-years and 2.7 million participants. Lancet (2011) 378(9785):31–40. 10.1016/S0140-6736(11)60679-X 21705069

[6] Review Team LaBrecque DR Abbas Z Anania F Ferenci P Khan AG World gastroenterology organisation global guidelines: nonalcoholic fatty liver disease and nonalcoholic steatohepatitis. J Clin Gastroenterol (2014) 48(6):467–73. 10.1097/MCG.0000000000000116 24921212

[7] Farzadfar F Finucane MM Danaei G Pelizzari PM Cowan MJ Paciorek CJ National, regional, and global trends in serum total cholesterol since 1980: systematic analysis of health examination surveys and epidemiological studies with 321 country-years and 3.0 million participants. Lancet (2011) 377(9765):578–86. 10.1016/S0140-6736(10)62038-7 21295847

[8] Schlegel A Gut P. Metabolic insights from zebrafish genetics, physiology, and chemical biology. Cell Mol Life Sci (2015) 72(12):2249–60. 10.1007/s00018-014-1816-8 25556679 PMC4439526

[9] Fang L Liu C Miller YI. Zebrafish models of dyslipidemia: relevance to atherosclerosis and angiogenesis. Transl Res (2014) 163(2):99–108. 10.1016/j.trsl.2013.09.004 24095954 PMC3946603

[10] Santoriello C Zon LI. Hooked! Modeling human disease in zebrafish. J Clin Invest (2012) 122(7):2337–43. 10.1172/JCI60434 22751109 PMC3386812

[11] Lieschke GJ Currie PD. Animal models of human disease: zebrafish swim into view. Nat Rev Genet (2007) 8(5):353–67. 10.1038/nrg2091 17440532

[12] MacRae CA Peterson RT. Zebrafish as tools for drug discovery. Nat Rev Drug Discov (2015) 14(10):721–31. 10.1038/nrd4627 26361349

[13] Kimura Y Hisano Y Kawahara A Higashijima S. Efficient generation of knock-in transgenic zebrafish carrying reporter/driver genes by CRISPR/Cas9-mediated genome engineering. Sci Rep (2014) 4:6545. 10.1038/srep06545 25293390 PMC4189020

[14] Auer TO Duroure K De Cian A Concordet JP Del Bene F. Highly efficient CRISPR/Cas9-mediated knock-in in zebrafish by homology-independent DNA repair. Genome Res (2014) 24(1):142–53. 10.1101/gr.161638.113 24179142 PMC3875856

[15] Hoshijima K Jurynec MJ Grunwald DJ Precise editing of the zebrafish genome made simple and efficient. Dev Cell (2016) 36(6):654–67. 10.1016/j.devcel.2016.02.015 27003937 PMC4806538

[16] Stamler J Wentworth D Neaton JD. Is relationship between serum cholesterol and risk of premature death from coronary heart disease continuous and graded? Findings in 356,222 primary screenees of the multiple risk factor intervention trial (MRFIT). JAMA (1986) 256(20):2823–8. 10.1001/jama.1986.03380200061022 3773199

[17] TG and HDL Working Group of the Exome Sequencing Project, National Heart, Lung, and Blood Institute. Loss-of-function mutations in APOC3, triglycerides, and coronary disease. N Engl J Med (2014) 371(1):22–31. 10.1056/NEJMoa1307095 24941081 PMC4180269

[18] JØrgensen AB Frikke-Schmidt R Nordestgaard BG Tybjaerg-Hansen A. Loss-of-function mutations in APOC3 and risk of ischemic vascular disease. N Engl J Med (2014) 371(1):32–41. 10.1056/NEJMoa1308027 24941082

[19] Babin PJ Gibbons GF The evolution of plasma cholesterol: direct utility or a “spandrel” of hepatic lipid metabolism? Prog Lipid Res (2009) 48(2):73–91. 10.1016/j.plipres.2008.11.002 19049814

[20] Young SG Zechner R. Biochemistry and pathophysiology of intravascular and intracellular lipolysis. Genes Dev (2013) 27(5):459–84. 10.1101/gad.209296.112 23475957 PMC3605461

[21] Conticello SG Thomas CJF Petersen-Mahrt SK Neuberger MS. Evolution of the AID/APOBEC family of polynucleotide (deoxy)cytidine deaminases. Mol Biol Evol (2005) 22(2):367–77. 10.1093/molbev/msi026 15496550

[22] Liu C Gates KP Fang L Amar MJ Schneider DA Geng H Apoc2 loss-of-function zebrafish mutant as a genetic model of hyperlipidemia. Dis Model Mech (2015) 8(8):989–98. 10.1242/dmm.019836 26044956 PMC4527288

[23] Otis JP Zeituni EM Thierer JH Anderson JL Brown AC Boehm ED Zebrafish as a model for apolipoprotein biology: comprehensive expression analysis and a role for ApoA-IV in regulating food intake. Dis Model Mech (2015) 8(3):295–309. 10.1242/dmm.018754 25633982 PMC4348566

[24] Marza E Barthe C Andre M Villeneuve L Helou C Babin PJ. Developmental expression and nutritional regulation of a zebrafish gene homologous to mammalian microsomal triglyceride transfer protein large subunit. Dev Dyn (2005) 232(2):506–18. 10.1002/dvdy.20251 15614773

[25] Schlegel A Stainier DY. Microsomal triglyceride transfer protein is required for yolk lipid utilization and absorption of dietary lipids in zebrafish larvae. Biochemistry (2006) 45(51):15179–87. 10.1021/bi0619268 17176039

[26] Avraham-Davidi I Ely Y Pham VN Castranova D Grunspan M Malkinson G ApoB-containing lipoproteins regulate angiogenesis by modulating expression of VEGF receptor 1. Nat Med (2012) 18(6):967–73. 10.1038/nm.2759 22581286 PMC3959651

[27] Levic DS Minkel JR Wang WD Rybski WM Melville DB Knapik EW. Animal model of Sar1b deficiency presents lipid absorption deficits similar to Anderson disease. J Mol Med (Berl) (2015) 93(2):165–76. 10.1007/s00109-014-1247-x 25559265 PMC4319984

[28] Zeituni EM Wilson MH Zheng X Iglesias PA Sepanski M Siddiqi MA Endoplasmic reticulum lipid flux influences enterocyte nuclear morphology and lipid-dependent transcriptional responses. J Biol Chem (2016) 291(45):23804–16. 10.1074/jbc.M116.749358 27655916 PMC5095432

[29] O’Hare EA Wang X Montasser ME Chang YP Mitchell BD Zaghloul NA. Disruption of ldlr causes increased LDL-c and vascular lipid accumulation in a zebrafish model of hypercholesterolemia. J Lipid Res (2014) 55(11):2242–53. 10.1194/jlr.M046540 25201834 PMC4617127

[30] Jin S Hong JH Jung SH Cho KH Turmeric and laurel aqueous extracts exhibit in vitro anti-atherosclerotic activity and in vivo hypolipidemic effects in a zebrafish model. J Med Food (2011) 14(3):247–56. 10.1089/jmf.2009.1389 21332404

[31] Hall J Qiu X. Structural and biophysical insight into cholesteryl ester-transfer protein. Biochem Soc Trans (2011) 39(4):1000–5. 10.1042/BST0391000 21787337

[32] Charles MA Kane JP. New molecular insights into CETP structure and function: a review. J Lipid Res (2012) 53(8):1451–8. 10.1194/jlr.R027011 22679067 PMC3540851

[33] von Eckardstein A. Implications of torcetrapib failure for the future of HDL therapy: is HDL-cholesterol the right target? Expert Rev Cardiovasc Ther (2010) 8(3):345–58. 10.1586/erc.10.6 20222814

[34] Stoletov K Fang L Choi S-H Hartvigsen K Hansen LF Hall C Vascular lipid accumulation, lipoprotein oxidation, and macrophage lipid uptake in hypercholesterolemic zebrafish. Circ Res (2009) 104(8):952–60. 10.1161/CIRCRESAHA.108.189803 19265037 PMC2834250

[35] Yin W Carballo-Jane E McLaren DG Mendoza VH Gagen K Geoghagen NS Plasma lipid profiling across species for the identification of optimal animal models of human dyslipidemia. J Lipid Res (2012) 53(1):51–65. 10.1194/jlr.M019927 22021650 PMC3243481

[36] Ishibashi S Brown MS Goldstein JL Gerard RD Hammer RE Herz J. Hypercholesterolemia in low density lipoprotein receptor knockout mice and its reversal by adenovirus-mediated gene delivery. J Clin Invest (1993) 92(2):883–93. 10.1172/JCI116663 8349823 PMC294927

[37] Ishibashi S Goldstein JL Brown MS Herz J Burns DK. Massive xanthomatosis and atherosclerosis in cholesterol-fed low density lipoprotein receptor-negative mice. J Clin Invest (1994) 93(5):1885–93. 10.1172/JCI117179 8182121 PMC294295

[38] Plump AS Smith JD Hayek T Aalto-Setala K Walsh A Verstuyft JG Severe hypercholesterolemia and atherosclerosis in apolipoprotein E-deficient mice created by homologous recombination in ES cells. Cell (1992) 71(2):343–53. 10.1016/0092-8674(92)90362-G 1423598

[39] Zhang SH Reddick RL Piedrahita JA Maeda N. Spontaneous hypercholesterolemia and arterial lesions in mice lacking apolipoprotein E. Science (1992) 258(5081):468–71. 10.1126/science.1411543 1411543

[40] de Silva HV Más-Oliva J Taylor JM Mahley RW. Identification of apolipoprotein B-100 low density lipoproteins, apolipoprotein B-48 remnants, and apolipoprotein E-rich high density lipoproteins in the mouse. J Lipid Res (1994) 35(7):1297–310. 7964191

[41] Getz GS Reardon CA Do the Apoe^−/−^ and Ldlr^−/−^ mice yield the same insight on atherogenesis? Arterioscler Thromb Vasc Biol (2016) 36(9):1734–41. 10.1161/atvbaha.116.306874 27386935 PMC5001905

[42] Najafi AH Aghili N Tilan JU Andrews JA Peng X Lassance-Soares RM A new murine model of stress-induced complex atherosclerotic lesions. Dis Model Mech (2013) 6(2):323–31. 10.1242/dmm.009977 23324329 PMC3597015

[43] Matoba T Sato K Egashira K. Mouse models of plaque rupture. Curr Opin Lipidol (2013) 24(5):419–25. 10.1097/MOL.0b013e3283646e4d 23942269

[44] Kaushik S Georga I Koumoundouros G Growth and body composition of zebrafish (*Danio rerio*) larvae fed a compound feed from first feeding onward: toward implications on nutrient requirements. Zebrafish (2011) 8(2):87–95. 10.1089/zeb.2011.0696 21663450

[45] Lawrence C The husbandry of zebrafish (*Danio rerio*): a review. Aquaculture (2007) 269(1–4):1–20. 10.1016/j.aquaculture.2007.04.077

[46] Oka T Nishimura Y Zang L Hirano M Shimada Y Wang Z Diet-induced obesity in zebrafish shares common pathophysiological pathways with mammalian obesity. BMC Physiol (2010) 10(1):21. 10.1186/1472-6793-10-21 20961460 PMC2972245

[47] Fang L Green SR Baek JS Lee S-H Ellett F Deer E In vivo visualization and attenuation of oxidized lipid accumulation in hypercholesterolemic zebrafish. J Clin Invest (2011) 121(12):4861–9. 10.1172/JCI57755 22105168 PMC3225997

[48] Fang L Harkewicz R Hartvigsen K Wiesner P Choi S-H Almazan F Oxidized cholesteryl esters and phospholipids in zebrafish larvae fed a high cholesterol diet. J Biol Chem (2010) 285(42):32343–51. 10.1074/jbc.M110.137257 20710028 PMC2952235

[49] Jin S Cho K-H. Water extracts of cinnamon and clove exhibits potent inhibition of protein glycation and anti-atherosclerotic activity in vitro and in vivo hypolipidemic activity in zebrafish. Food Chem Toxicol (2011) 49(7):1521–9. 10.1016/j.fct.2011.03.043 21443916

[50] Kim JY Hong JH Jung HK Jeong YS Cho KH. Grape skin and loquat leaf extracts and acai puree have potent anti-atherosclerotic and anti-diabetic activity in vitro and in vivo in hypercholesterolemic zebrafish. Int J Mol Med (2012) 30(3):606–14. 10.3892/ijmm.2012.1045 22751734

[51] Kim J-Y Seo J Cho K-H. Aspartame-fed zebrafish exhibit acute deaths with swimming defects and saccharin-fed zebrafish have elevation of cholesteryl ester transfer protein activity in hypercholesterolemia. Food Chem Toxicol (2011) 49(11):2899–905. 10.1016/j.fct.2011.08.001 21855599

[52] Kim J-Y Park K-H Kim J Choi I Cho K-H. Modified high-density lipoproteins by artificial sweetener, aspartame, and saccharin, showed loss of anti-atherosclerotic activity and toxicity in zebrafish. Cardiovasc Toxicol (2015) 15(1):79–89. 10.1007/s12012-014-9273-z 25142179

[53] Suez J Korem T Zeevi D Zilberman-Schapira G Thaiss CA Maza O Artificial sweeteners induce glucose intolerance by altering the gut microbiota. Nature (2014) 514(7521):181–6. 10.1038/nature13793 25231862

[54] Nicholls SJ Brewer HB Kastelein JJ Krueger KA Wang MD Shao M Effects of the CETP inhibitor evacetrapib administered as monotherapy or in combination with statins on HDL and LDL cholesterol: a randomized controlled trial. JAMA (2011) 306(19):2099–109. 10.1001/jama.2011.1649 22089718

[55] Hovingh GK Kastelein JJP van Deventer SJH Round P Ford J Saleheen D Cholesterol ester transfer protein inhibition by TA-8995 in patients with mild dyslipidaemia (TULIP): a randomised, double-blind, placebo-controlled phase 2 trial. Lancet (2011) 386(9992):452–60. 10.1016/S0140-6736(15)60158-1 26047975

[56] Baggio G Manzato E Gabelli C Fellin R Martini S Enzi GB Apolipoprotein C-II deficiency syndrome. Clinical features, lipoprotein characterization, lipase activity, and correction of hypertriglyceridemia after apolipoprotein C-II administration in two affected patients. J Clin Invest (1986) 77(2):520–7. 10.1172/JCI112332 3944267 PMC423374

[57] Calkin AC Tontonoz P. Transcriptional integration of metabolism by the nuclear sterol-activated receptors LXR and FXR. Nat Rev Mol Cell Biol (2012) 13(4):213–24. 10.1038/nrm3312 22414897 PMC3597092

[58] Uhlen M Fagerberg L Hallstrom BM Lindskog C Oksvold P Mardinoglu A Proteomics. Tissue-based map of the human proteome. Science (2015) 347(6220):1260419 10.1126/science.1260419 25613900

[59] Repa JJ Liang G Ou J Bashmakov Y Lobaccaro JM Shimomura I Regulation of mouse sterol regulatory element-binding protein-1c gene (SREBP-1c) by oxysterol receptors, LXRalpha and LXRbeta. Genes Dev (2000) 14(22):2819–30. 10.1101/gad.844900 11090130 PMC317055

[60] Schultz JR Tu H Luk A Repa JJ Medina JC Li L Role of LXRs in control of lipogenesis. Genes Dev (2000) 14(22):2831–8. 10.1101/gad.850400 11090131 PMC317060

[61] Kirchgessner TG Sleph P Ostrowski J Lupisella J Ryan CS Liu X Beneficial and adverse effects of an LXR agonist on human lipid and lipoprotein metabolism and circulating neutrophils. Cell Metab (2016) 24(2):223–33. 10.1016/j.cmet.2016.07.016 27508871

[62] Cruz-Garcia L Schlegel A. Lxr-driven enterocyte lipid droplet formation delays transport of ingested lipids. J Lipid Res (2014) 55(9):1944–58. 10.1194/jlr.M052845 25030662 PMC4617358

[63] Cronan MR Beerman RW Rosenberg AF Saelens JW Johnson MG Oehlers SH Macrophage epithelial reprogramming underlies mycobacterial granuloma formation and promotes infection. Immunity (2016) 45(4):861–76. 10.1016/j.immuni.2016.09.014 27760340 PMC5268069

[64] Chen Z Hu Y Cumming BM Lu P Feng L Deng J Mycobacterial WhiB6 differentially regulates ESX-1 and the Dos Regulon to modulate granuloma formation and virulence in zebrafish. Cell Rep (2016) 16(9):2512–24. 10.1016/j.celrep.2016.07.080 27545883

[65] Berg RD Levitte S O’Sullivan MP O’Leary SM Cambier CJ Cameron J Lysosomal disorders drive susceptibility to tuberculosis by compromising macrophage migration. Cell (2016) 165(1):139–52. 10.1016/j.cell.2016.02.034 27015311 PMC4819607

[66] Gut P Baeza-Raja B Andersson O Hasenkamp L Hsiao J Hesselson D Whole-organism screening for gluconeogenesis identifies activators of fasting metabolism. Nat Chem Biol (2013) 9(2):97–104. 10.1038/nchembio.1136 23201900 PMC3552031

[67] Weger BD Weger M Nusser M Brenner-Weiss G Dickmeis T. A chemical screening system for glucocorticoid stress hormone signaling in an intact vertebrate. ACS Chem Biol (2012) 7(7):1178–83. 10.1021/cb3000474 22545806 PMC3401037

[68] Global Lipids Genetics Consortium Willer CJ Schmidt EM Sengupta S Peloso GM Gustafsson S Discovery and refinement of loci associated with lipid levels. Nat Genet (2013) 45(11):1274–83. 10.1038/ng.2797 24097068 PMC3838666

[69] Karanth S Zinkhan EK Hill JT Yost HJ Schlegel A. FOXN3 regulates hepatic glucose utilization. Cell Rep (2016) 15(2):2745–55. 10.1016/j.celrep.2016.05.056 27292639 PMC4917433

[70] Kettleborough RN Busch-Nentwich EM Harvey SA Dooley CM de Bruijn E van Eeden F A systematic genome-wide analysis of zebrafish protein-coding gene function. Nature (2013) 496(7446):494–7. 10.1038/nature11992 23594742 PMC3743023

